# Author Correction: GWAS for systemic sclerosis identifies six novel susceptibility loci including one in the Fcγ receptor region

**DOI:** 10.1038/s41467-026-68405-4

**Published:** 2026-01-19

**Authors:** Yuki Ishikawa, Nao Tanaka, Yoshihide Asano, Masanari Kodera, Yuichiro Shirai, Mitsuteru Akahoshi, Minoru Hasegawa, Takashi Matsushita, Kazuyoshi Saito, Sei-ichiro Motegi, Hajime Yoshifuji, Ayumi Yoshizaki, Tomohiro Kohmoto, Kae Takagi, Akira Oka, Miho Kanda, Yoshihito Tanaka, Yumi Ito, Kazuhisa Nakano, Hiroshi Kasamatsu, Akira Utsunomiya, Akiko Sekiguchi, Hiroaki Niiro, Masatoshi Jinnin, Katsunari Makino, Takamitsu Makino, Hironobu Ihn, Motohisa Yamamoto, Chisako Suzuki, Hiroki Takahashi, Emi Nishida, Akimichi Morita, Toshiyuki Yamamoto, Manabu Fujimoto, Yuya Kondo, Daisuke Goto, Takayuki Sumida, Naho Ayuzawa, Hidetoshi Yanagida, Tetsuya Horita, Tatsuya Atsumi, Hirahito Endo, Yoshihito Shima, Atsushi Kumanogoh, Jun Hirata, Nao Otomo, Hiroyuki Suetsugu, Yoshinao Koike, Kohei Tomizuka, Soichiro Yoshino, Xiaoxi Liu, Shuji Ito, Keiko Hikino, Akari Suzuki, Yukihide Momozawa, Shiro Ikegawa, Yoshiya Tanaka, Osamu Ishikawa, Kazuhiko Takehara, Takeshi Torii, Shinichi Sato, Yukinori Okada, Tsuneyo Mimori, Fumihiko Matsuda, Koichi Matsuda, Tiffany Amariuta, Issei Imoto, Keitaro Matsuo, Masataka Kuwana, Yasushi Kawaguchi, Koichiro Ohmura, Chikashi Terao

**Affiliations:** 1 RIKEN Center for Integrative Medical Sciences, The Laboratory for Statistical and Translational Genetics, Yokohama, Japan; 2 Department of Rheumatology, Graduate School of Medical and Dental Sciences, Tokyo Medical and Dental University, Tokyo, Japan; 3 Department of Dermatology, Tohoku University Graduate School of Medicine, Sendai, Japan; 4 Department of Dermatology, The University of Tokyo, Tokyo, Japan; 5 Department of Dermatology, Chukyo Hospital, Japan Community Health Care Organization, Nagoya, Japan; 6 Department of Allergy and Rheumatology, Nippon Medical School Graduate School of Medicine, Tokyo, Japan; 7 Department of Medicine and Biosystemic Science, Kyushu University Graduate School of Medical Sciences, Fukuoka, Japan; 8 Department of Rheumatology, Saga University Hospital, Saga, Japan; 9 Faculty of Medical Sciences, Department of Dermatology, University of Fukui, Fukui, Japan; 10 Department of Dermatology, Faculty of Medicine, Institute of Medical, Pharmaceutical and Health Sciences, Kanazawa University, Kanazawa, Japan; 11 The First Department of Internal Medicine, University of Occupational and Environmental Health, Japan, Kitakyushu, Japan; 12 Department of Dermatology, Gunma University Graduate School of Medicine, Maebashi, Japan; 13 Department of Rheumatology and Clinical Immunology, Graduate School of Medicine, Kyoto University, Kyoto, Japan; 14 Aichi Cancer Center Research Institute, Division of Molecular Genetics, Nagoya, Japan; 15 Tokyo Women’s Medical University, Adachi Medical Center, Tokyo, Japan; 16 Department of Molecular Life Sciences, Division of Basic Medical Science and Molecular Medicine, Tokai University School of Medicine, Isehara, Japan; 17 Department of Dermatology, Wakayama Medical University Graduate School of Medicine, Wakayama, Japan; 18 Department of Dermatology and Plastic Surgery, Faculty of Life Sciences, Kumamoto University, Kumamoto, Japan; 19 Department of Rheumatology and Allergy, IMSUT Hospital, The Institute of Medical Science, The University of Tokyo, Tokyo, Japan; 20 Department of Rheumatology and Clinical Immunology, Sapporo Medical University School of Medicine, Sapporo, Japan; 21 Department of Geriatric and Environmental Dermatology, Nagoya City University Graduate School of Medical Sciences, Nagoya, Japan; 22 Department of Dermatology, Okazaki City Hospital, Okazaki, Japan; 23 Department of Dermatology, Fukushima Medical University, School of Medicine, Fukushima, Japan; 24 Department of Dermatology, Graduate School of Medicine, Osaka University, Osaka, Japan; 25 Department of Rheumatology, Institute of Medicine, University of Tsukuba, Tsukuba, Japan; 26 Department of Clinical Immunology, National Hospital Organization, Utano National Hospital, Kyoto, Japan; 27 Faculty of Medicine and Graduate School of Medicine, Department of Rheumatology, Endocrinology and Nephrology, Hokkaido University, Sapporo, Japan; 28 Omori Medical Center, Toho University, Rheumatic Disease Center, Tokyo, Japan; 29 Department of Respiratory Medicine and Clinical Immunology, Osaka University Graduate School of Medicine, Osaka, Japan; 30 Immunology Frontier Center, Osaka University, Statistical Immunology, Osaka, Japan; 31 RIKEN Center for Integrative Medical Sciences, The Laboratory for Pharmacogenomics, Yokohama, Japan; 32 RIKEN Center for Integrative Medical Sciences, The Laboratory for Autoimmune Diseases, Yokohama, Japan; 33 RIKEN Center for Integrative Medical Sciences, The Laboratory for Genotyping Development, Yokohama, Japan; 34 RIKEN Center for Integrative Medical Sciences, The Laboratory for Bone and Joint Diseases, Yokohama, Japan; 35 Torii Clinic, Maizuru, Japan; 36 Ijinkai Takeada General Hospital, Kyoto, Japan; 37 Graduate School of Medicine, Kyoto University, Center for Genomic Medicine, Kyoto, Japan; 38 Institute of Medical Science, The University of Tokyo, Laboratory of Genome Technology, Human Genome Center, Tokyo, Japan; 39 Department of Computational Biology and Medical Sciences, Laboratory of Clinical Genome Sequencing, Graduate School of Frontier Sciences, The University of Tokyo, Tokyo, Japan; 40 Center for Data Sciences, Harvard Medical School, Boston, MA USA; 41 Divisions of Genetics and Rheumatology, Department of Medicine, Brigham and Women’s Hospital, Harvard Medical School, Boston, MA USA; 42 Program in Medical and Population Genetics, Broad Institute of MIT and Harvard, Cambridge, MA USA; 43 Department of Biomedical Informatics, Harvard Medical School, Boston, MA USA; 44 Graduate School of Arts and Sciences, Harvard University, Cambridge, MA USA; 45 Aichi Cancer Center Research Institute, Nagoya, Japan; 46 Aichi Cancer Center Research Institute, Division of Cancer Epidemiology and Prevention, Nagoya, Japan; 47 Tokyo Women’s Medical University, Division of Rheumatology, Department of Internal Medicine, Tokyo, Japan; 48 Shizuoka General Hospital, The Clinical Research Center, Shizuoka, Japan; 49 The Department of Applied Genetics, School of Pharmaceutical Sciences, University of Shizuoka, Shizuoka, Japan

**Keywords:** Genome-wide association studies, Immunogenetics, Systemic sclerosis, Gene regulation, Gene expression

Correction to: *Nature Communications* 10.1038/s41467-023-44541-z, published online 31 January 2024

In the version of the article initially published, due to a conversion error, values in the Table 1 beta coefficient of logistic regression “BETA” columns were incorrect. For comparison, the original and revised table versions are available as Supplementary Information accompanying this amendment. In the Supplementary Table 14 reference and aleternatice allele columns, data were missing and are now included. The tables are amended in the HTML and PDF versions of the article.

## Supplementary information

Original and revised Table 1

