# Supplementary material for: GWAS for systemic sclerosis identifies six novel susceptibility loci including one in the Fcγ receptor region
Source: Nat Commun. 2024 Jan 31;15:319. doi: 10.1038/s41467-023-44541-z (PMC10830486; doi:10.1038/s41467-023-44541-z)
Supplement: Supplementary file 1 — Supplementary Information [file 41467_2023_44541_MOESM1_ESM.pdf]

Supplementary Information

GWAS for systemic sclerosis identified six novel susceptibility loci including one in the Fcγ receptor region

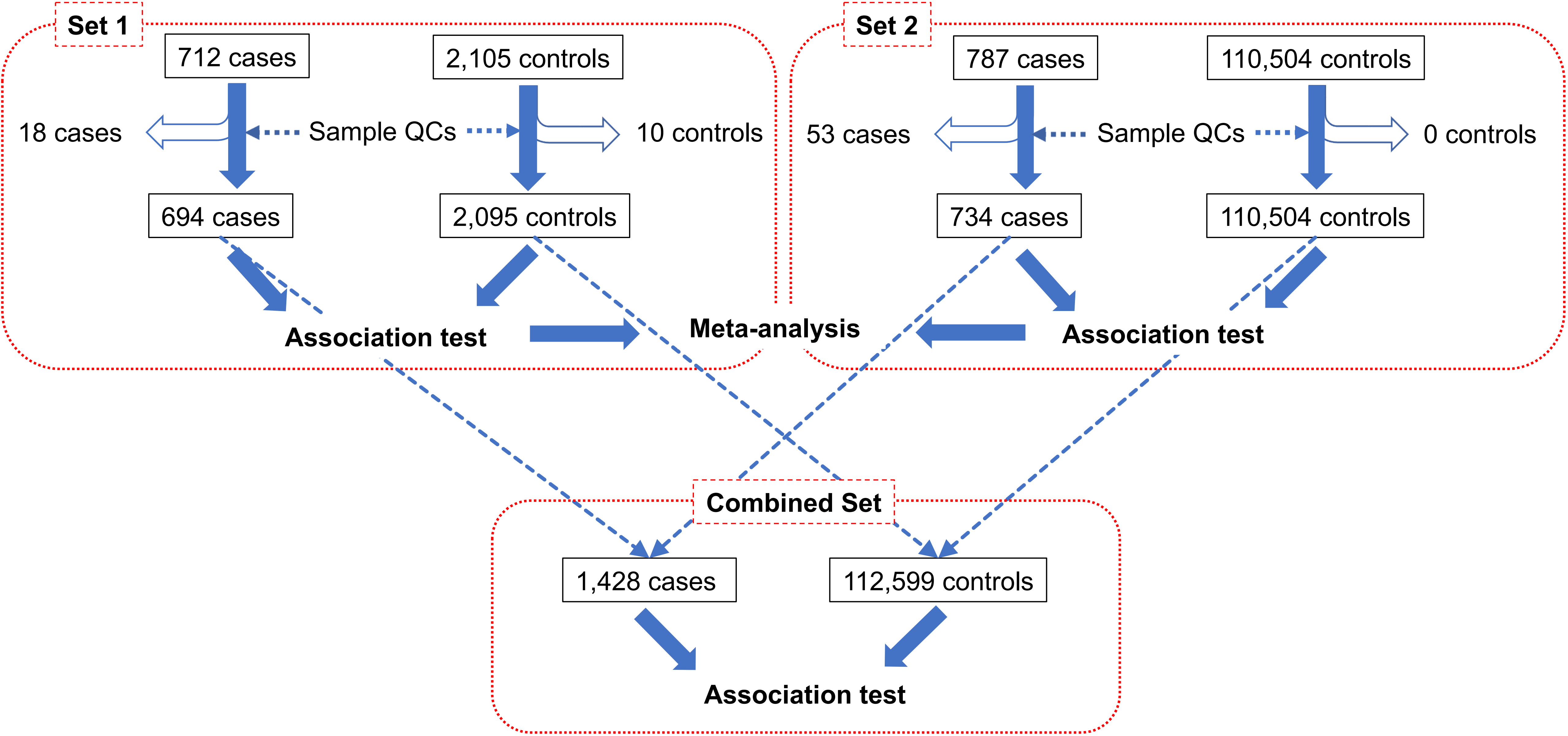

Supplementary Figure. 1 A workflow of sample quality controls, association tests, and meta-analysis for the Japanese subjects in the present study.
Set 1 consisted of 712 cases enrolled in the previous study and 2,105 controls. Set 2 consisted of newly enrolled 787 cases and 110,504 controls enrolled from the BioBank Japan project. Indicated numbers of subjects were excluded after sample quality controls (QCs) leaving 694 cases and 2,095 controls in Set 1 and 734 cases and 110,504 controls in Set 2. Association tests were conducted for each set and the results were meta-analyzed. Set 1 and Set 2 were combined to generate the combined dataset consisting of 1,428 cases and 112,599 controls and an association test was conducted.

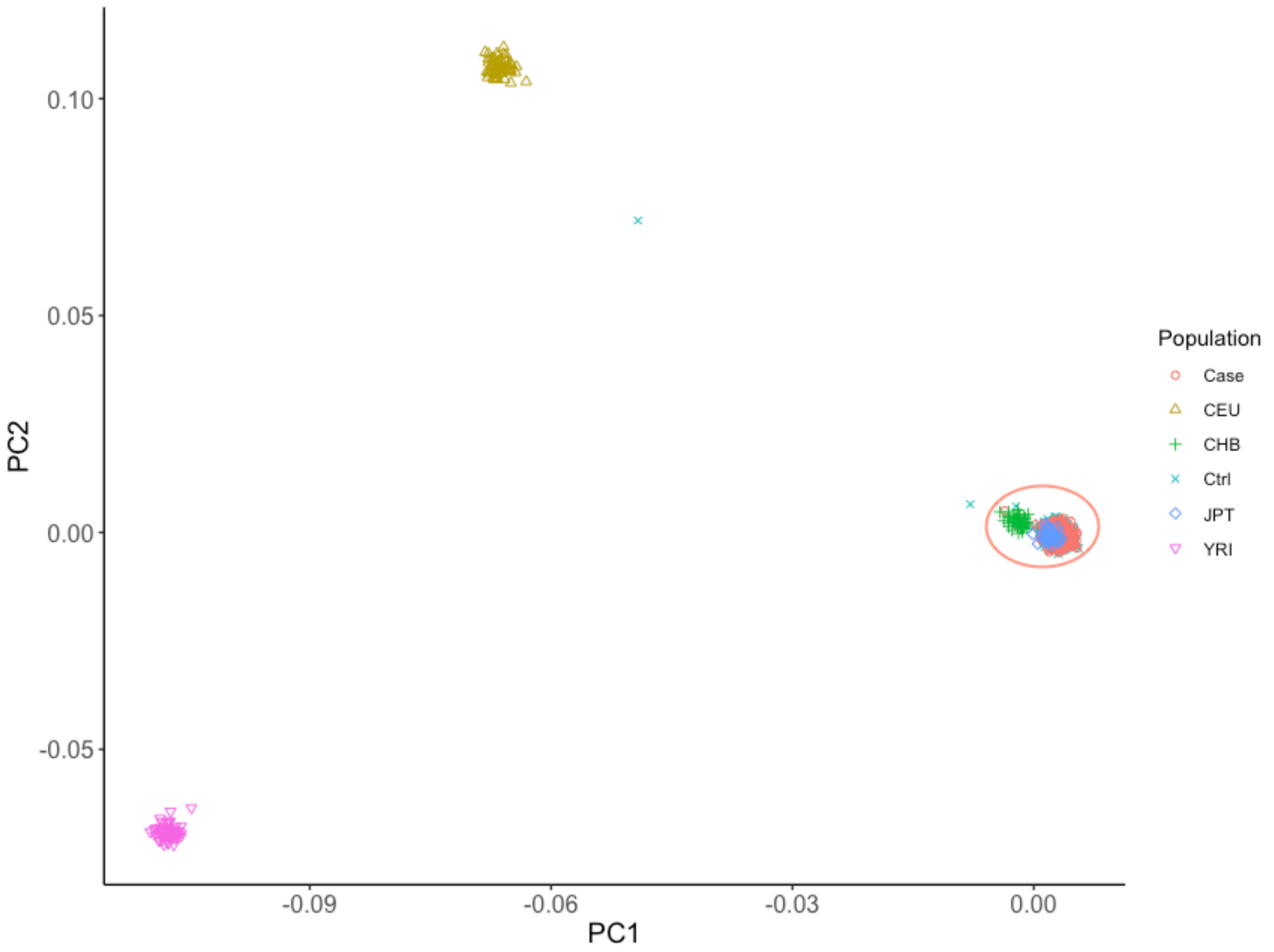

Supplementary Fig. 2 Principal component analysis for population stratification
Samples in the present study (1,428 Case and 112,599 Ctrl) were subjected to principal component (PC) analysis based on the four populations, YRI, CEU, CHB, and JPT. The samples plotted outside the red circle, representing the East Asian cluster, were excluded from the subsequent analyses. YRI, Yoruba in Ibadan, Nigeria (n=60); CEU, Utah residents with Northern and Western European ancestry from the CEPH collection (n=60); CHB, Han Chinese in Beijing, China (n=45); JPT, Japanese in Tokyo, Japan (n=44). Source data are provided as a Source Data file.

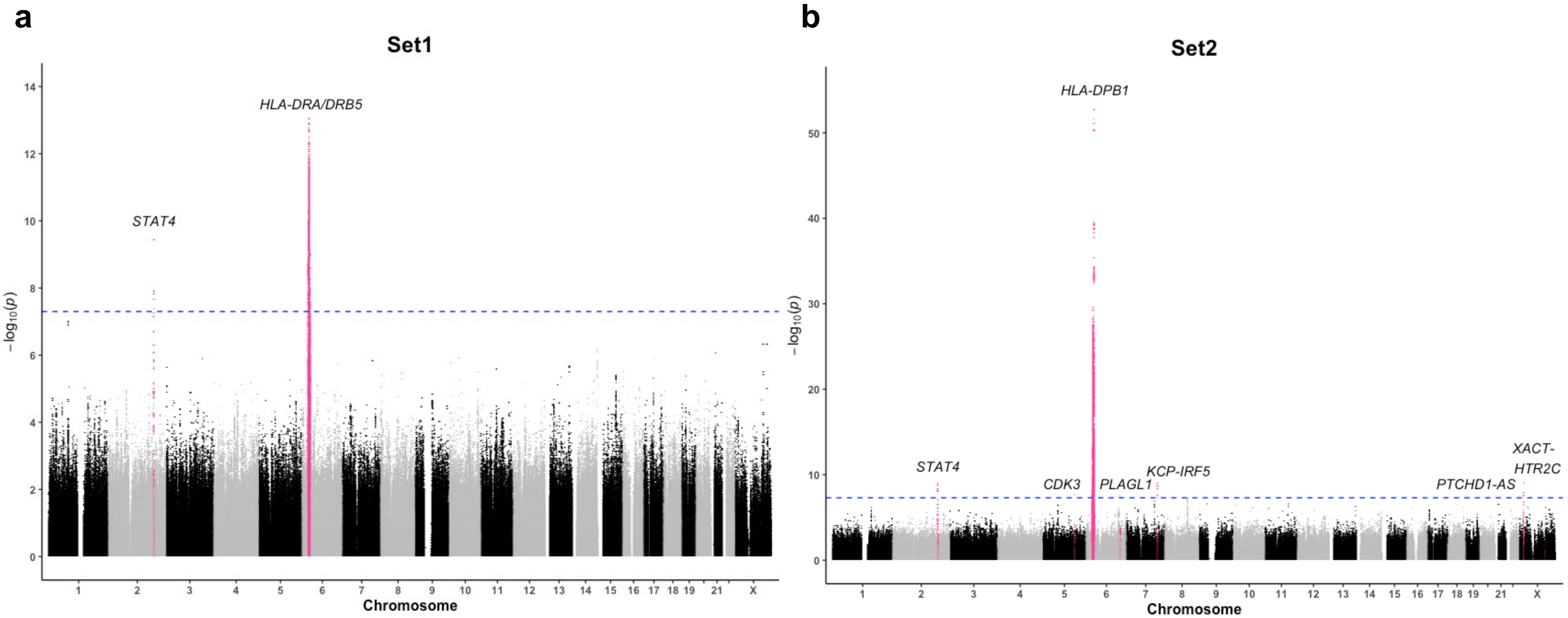

Supplementary Fig. 3 Manhattan plots of association tests for Set 1 and Set 2 datasets
Manhattan plots of association tests by logistic regression for Set 1 (a) (694 cases and 2,095 controls) and Set 2 (b) (734 cases and 110,504 controls) of Japanese samples are presented. Genome-wide significant ($p=5\times10^{-8}$) threshold is presented by a blue dot line. Source data are provided as a Source Data file.

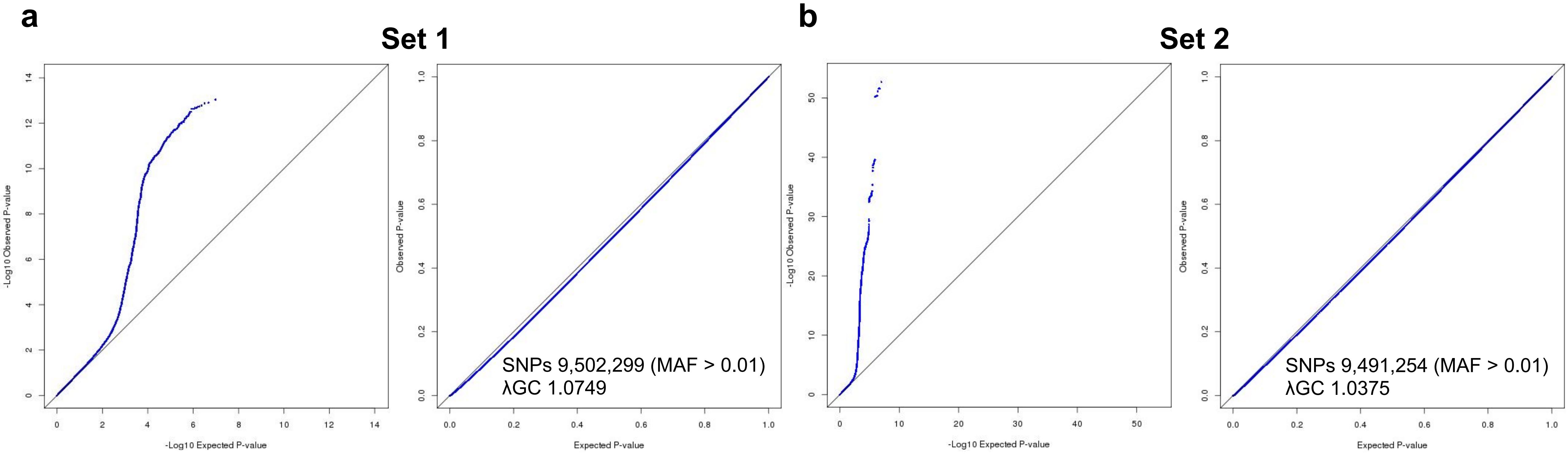

Supplementary Fig. 4 Quantile-quantile plots of association tests for Set 1 and Set 2.
Quartile-quartile plots (QQ plots) of association tests by logistic regression for Set 1 (a) (694 cases and 2,095 controls) and Set 2 (b) (734 cases and 110,504 controls) of the Japanese samples are presented. The numbers of single nucleotide polymorphisms (SNPs) included in the analyses and genomic inflation factors (λGC) are presented in the boxes right below the graphs. MAF, minor allele frequency. Source data are provided as a Source Data file.

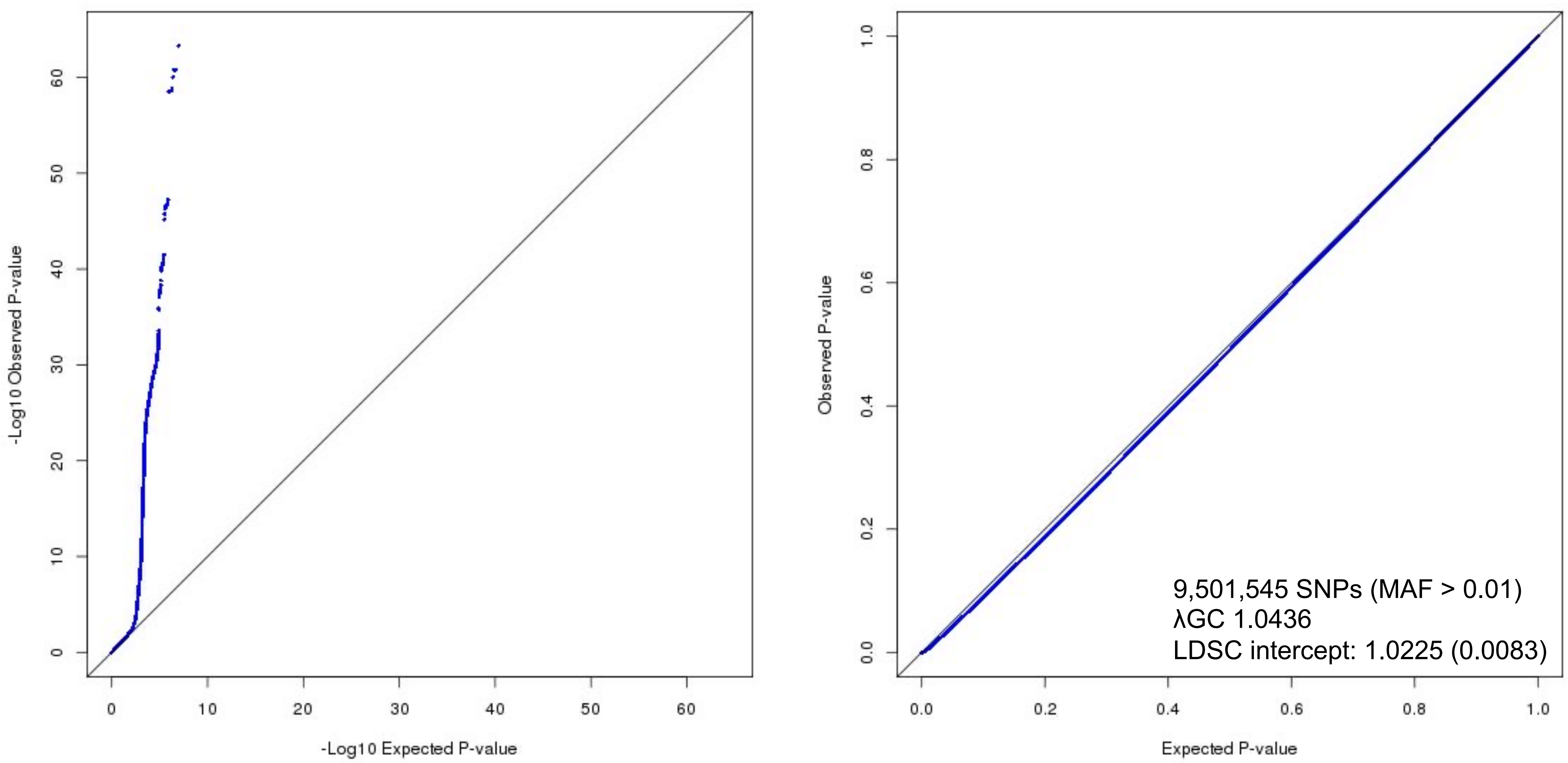

Supplementary Fig. 5 Quantile-quantile plot of the association test for a combined Japanese dataset.
Quartile-quartile plots (Q-Q plots) of an association test by logistic regression for a combined Japanese dataset (1,428 cases and 112,599 controls) are presented. Data are plotted in -log10 (p-values) (left) or in natural numbers (right). The number of SNPs with minor allele frequency (MAF) > 0.01 included, a genomic inflation factor (λGC), and an intercept of linkage disequilibrium score regression (LDSC) are presented at the bottom of the right panel. Source data are provided as a Source Data file.

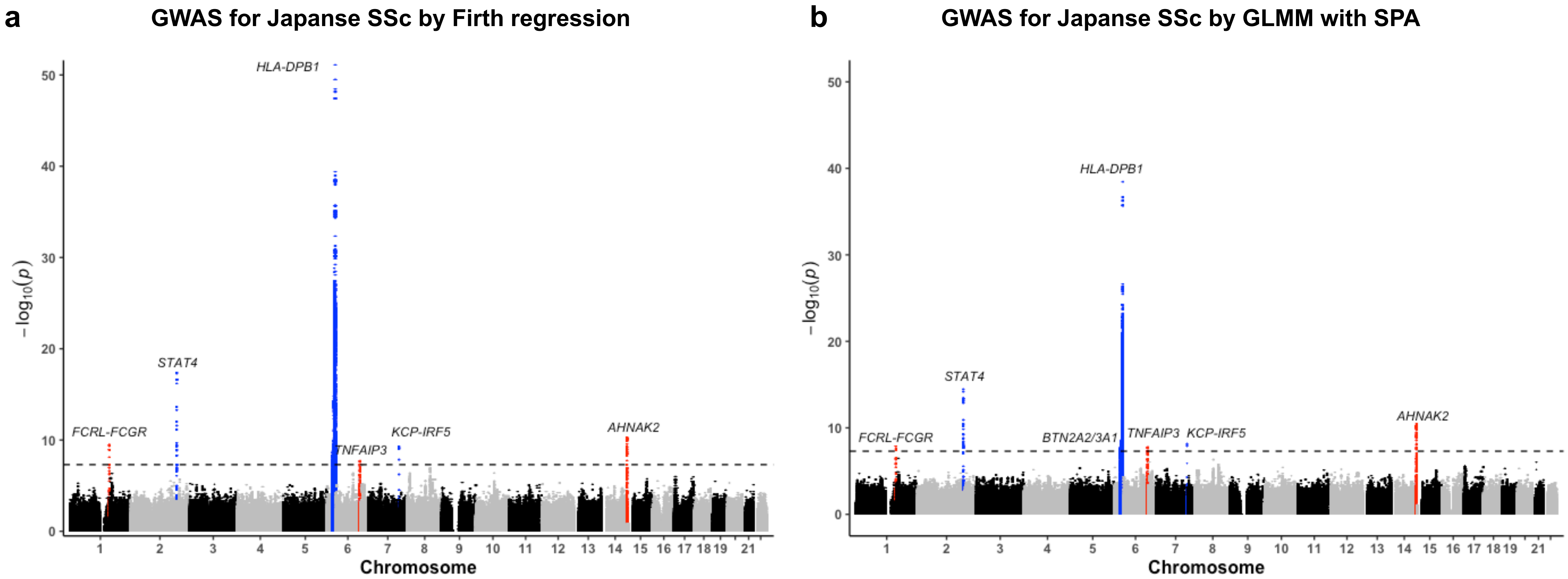

Supplementary Fig. 6 Manhattan plots of the association tests for a combined Japanese dataset by the firth regression and a generalized mixed model with the saddle point approximation.

Manhattan plots of association tests by the firth regression (a) (1,428 cases and 13,946 controls) and a generalized linear mixed model (GLMM) with the saddle point approximation (SPA) (b) (1,428 cases and 112,599 controls) are presented. Genome-wide significant ($p=5\times10^{-8}$) threshold is presented by a blue dotted line. Source data are provided as a Source Data file.

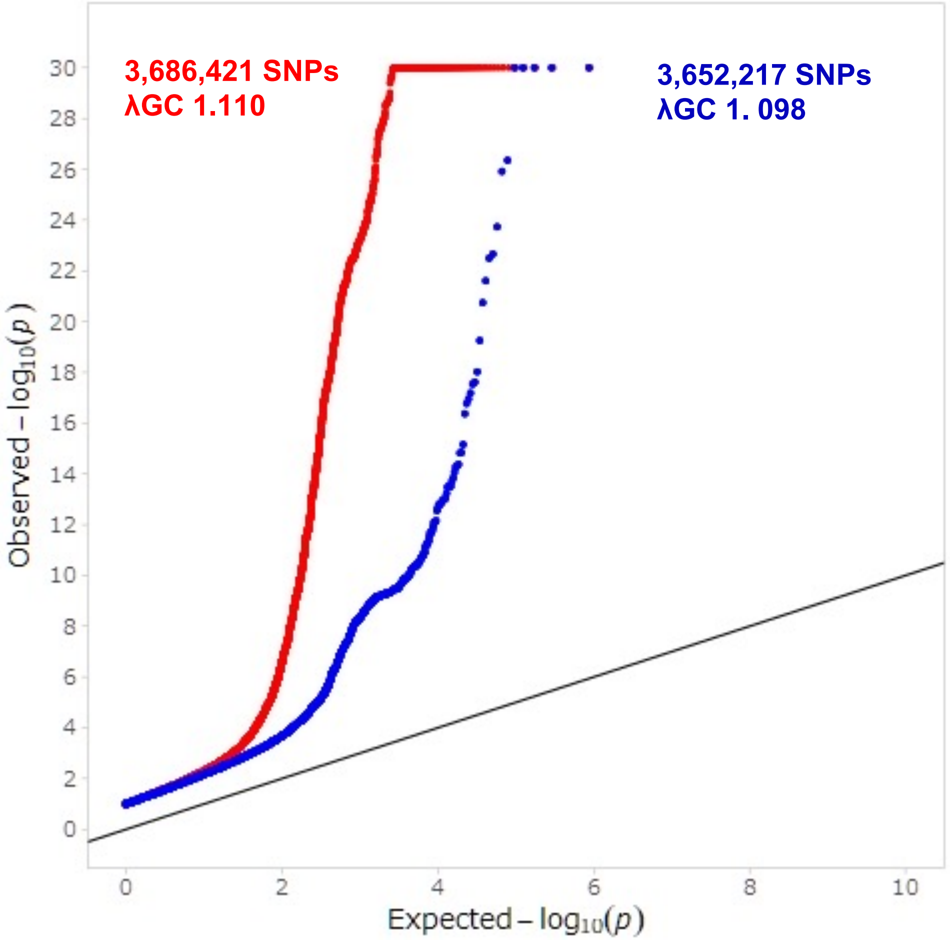

Supplementary Fig. 7 Quantile-quantile (QQ) plot of a meta-analysis for the European and the Japanese GWASs
The Q-Q plots of the trans-ethnic genome-wide association study (GWAS) for European (9,095 cases and 17,584 controls) and Japanese (1,428 cases and 112,599 controls) GWAS of systemic sclerosis with (red) or without (blue) SNPs in the HLA region. Source data are provided as a Source Data file.

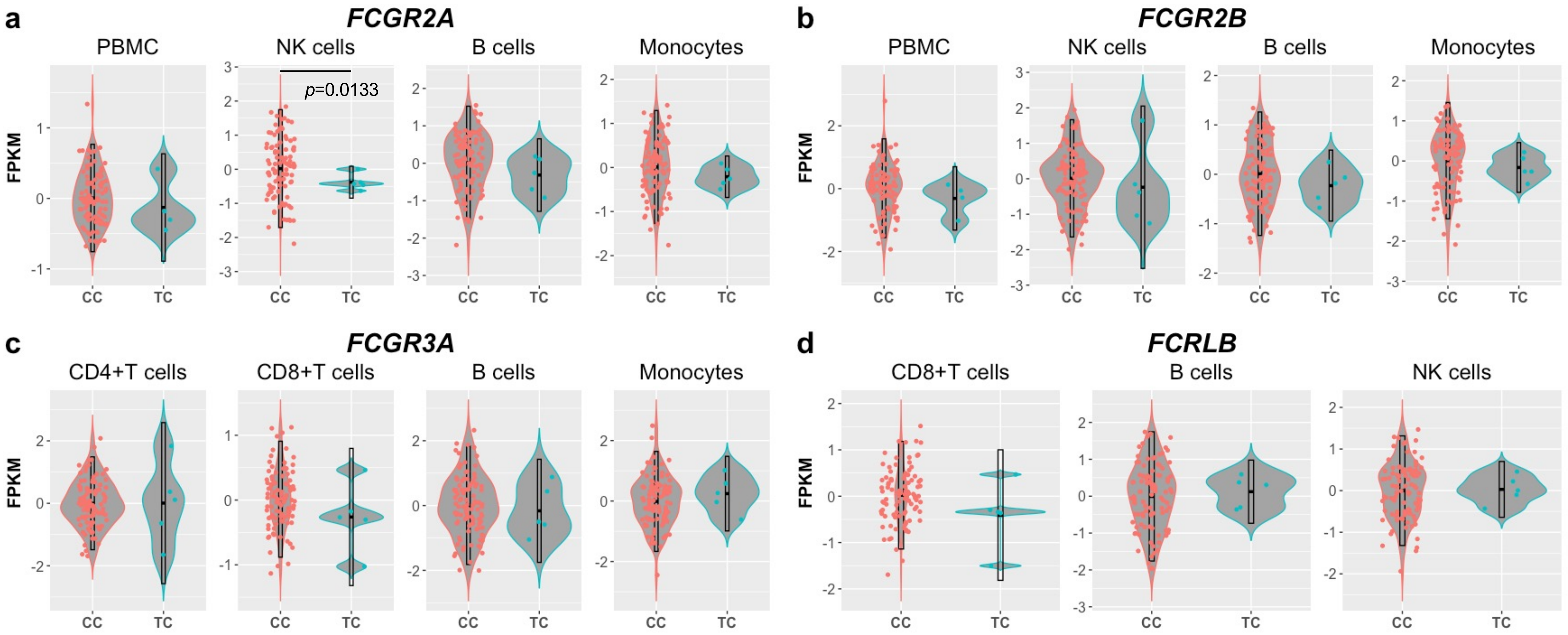

Supplementary Fig. 8 Associations of rs10917688 with the expression of *FCGR2A*, *FCGR2B*, *FCGR3A*, and *FCRLB*.
The expression of *FCGR2A* (a), *FCGR2B* (b), *FCGR3A* (c), and *FCRLB* (d) in the different cell types by rs10917688 genotypes are presented. The difference of expression between genotypes was compared by unpaired t-tests and an only significant p-value ($p<0.05$) is presented. Genotype information was extracted from the Japanese expression quantitative trait (eQTL) dataset of six white blood cell subpopulations (N=105; N=100 for CC and N=5 for TC). C and T are the reference allele and the alternative allele, respectively. There were no subjects with homozygous for the alternative allele (TT). Each box plot indicates a mean and standard deviations. Source data are provided as a Source Data file.

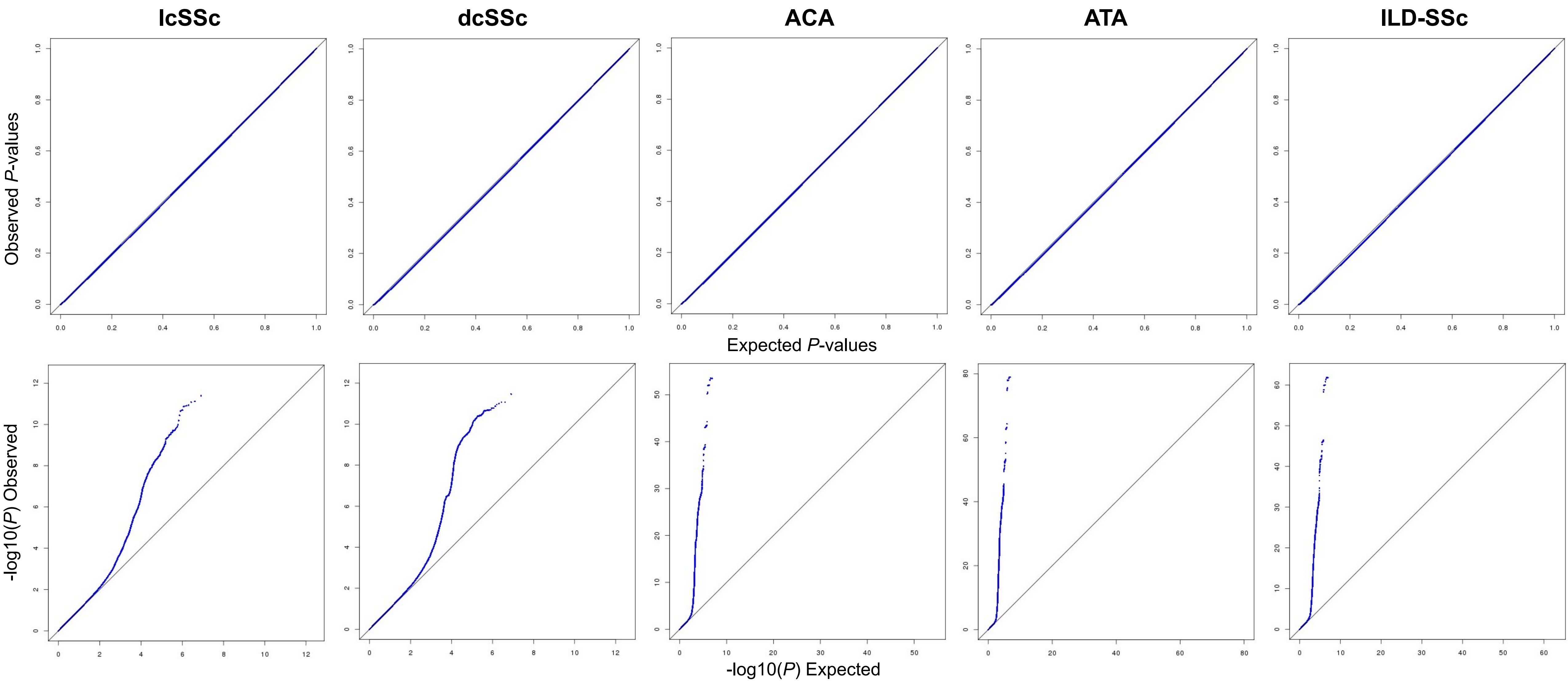

Case number = 679
SNPs 8,104,349 (MAF $\geq$ 0.01)
λGC 1.027

Case number = 575
SNPs 8,104,285 (MAF $\geq$ 0.01)
λGC 1.020

Case number = 429
SNPs 8,104,342 (MAF $\geq$ 0.01)
λGC 1.032

Case number = 463
SNPs 8,104,353 (MAF $\geq$ 0.01)
λGC 1.031

Case number = 625
SNPs 8,104,378 (MAF $\geq$ 0.01)
λGC 1.037

Supplementary Fig. 9 Q-Q plots of association tests for major clinical subtypes of SSc.
Quantile-quantile (Q-Q) plots of association tests by logistic regression for systemic sclerosis (SSc) subtypes are presented. Case numbers, numbers of single nucleotide polymorphisms (SNPs) utilized in the analyses, and genomic inflation factors were indicated in the boxes below. The sample size of control is 112,599. Source data are provided as a Source Data file.

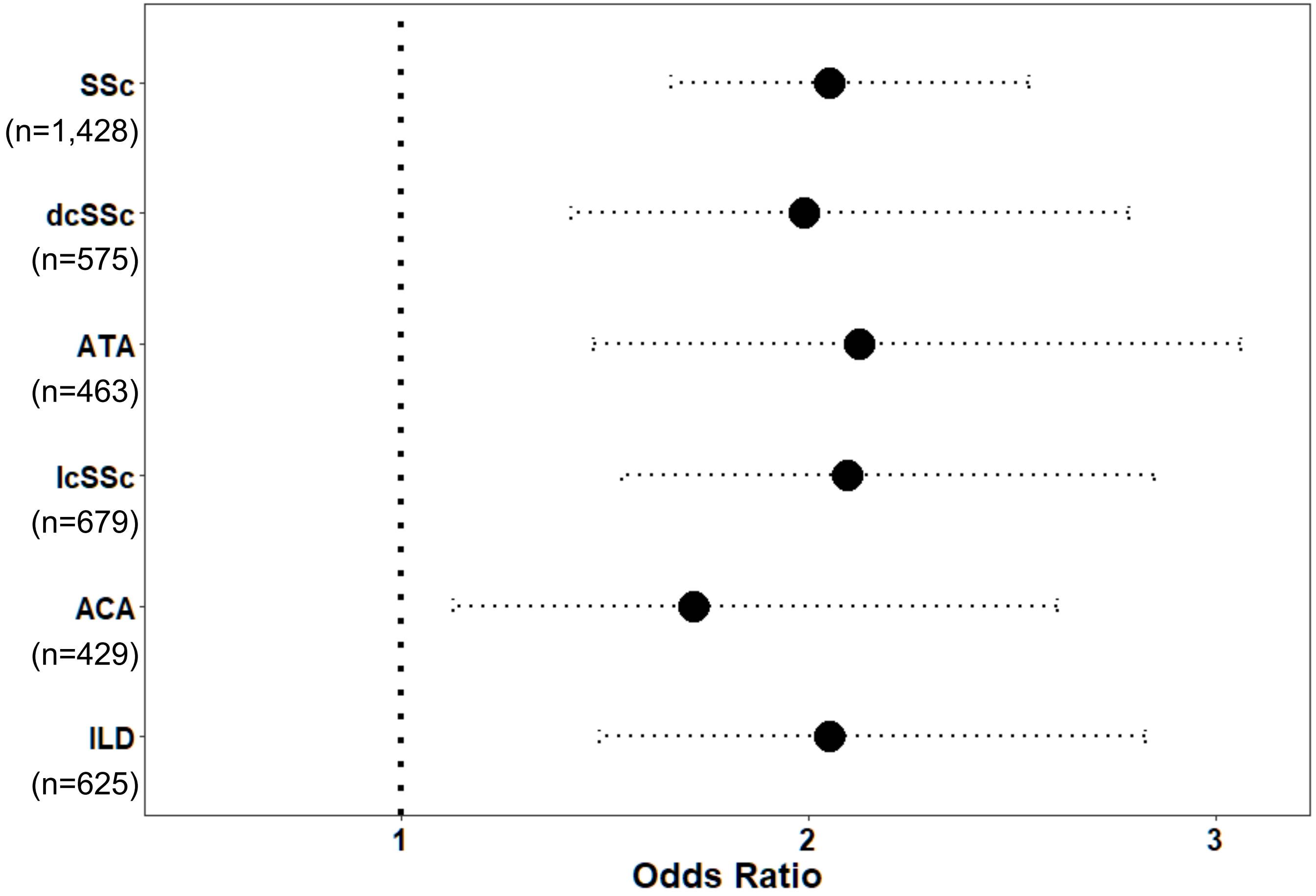

Supplementary Fig. 10 Comparable risk of rs6697139 among major subtypes of SSc except for the ACA-positive subset.

Odd ratios (black dots) with 95% confidence intervals (dotted bars) of rs6697139 in association tests for an entire SSc and the major clinical subtypes are presented. SSc, systemic sclerosis; lcSSc, limited cutaneous SSc; dcSSc**,** diffuse cutaneous SSc; ATA anti-topoisomerase I antibody; ACA, anti-centromere antibody; ILD, interstitial lung disease. Source data are provided as a Source Data file.
