# Supplementary material for: GWAS for systemic sclerosis identifies six novel susceptibility loci including one in the Fcγ receptor region
Source: Nat Commun. 2024 Jan 31;15:319. doi: 10.1038/s41467-023-44541-z (PMC10830486; doi:10.1038/s41467-023-44541-z)
Supplement: Supplementary file 3 — Description of Additional Supplementary Files [file 41467_2023_44541_MOESM3_ESM.pdf]

Description of Additional Supplementary Files:

Supplementary Data 1: The summary data of the previous GWASs and the present GWAS for 26 known risk loci.

Supplementary Data 2: Genome-wide significant SNPs identified by GWAS of Japanese Set 1 dataset.

Supplementary Data 3: Genome-wide significant SNPs identified by GWAS of Japanese Set 2 dataset.

Supplementary Data 4: Gene annotations by ANNOVAR for the lead SNPs identified by GWAS of Japanese SSc and their LD SNPs (r2 > 0.8).

Supplementary Data 5: Loss of function prediction by LossOf-Function Transcript Effect Estimator (LOFTEE) for the lead SNPs identified by GWAS of Japanese SSc and their LD SNPs (r2 > 0.8).

Supplementary Data 6: Functional impacts of a single amino acid replacement predicted by polyphen2 for the lead SNPs identified by GWAS of Japanese SSc and their LD SNPs (r2 > 0.8).

Supplementary Data 7: Functional impacts of a single amino acid replacement predicted by SIFT for the lead SNPs identified by GWAS of Japanese SSc and their LD SNPs (r2 > 0.8).

Supplementary Data 8: 95% credible sets identified by finemapping for the Japanese GWAS

Supplementary Data 9: Significantly associated genetissue/cell pairs identified by TWAS for Japanese SSc.

Supplementary Data 10: The output of gene set analyses by FUMA.

Supplementary Data 11: The output of tans-ethnic genetic correlation analysis by Popcorn.

Supplementary Data 12: The result of trans-ethnic metaanalysis for the lead SNPs identified in GWAS of Japanese SSc.

Supplementary Data 13: 95% credible sets of fine-mapped SNPs for the trans-ethnic meta-GWAS.

Supplementary Data 14: The 95% credible sets identified by fine-mapping for the European meta-GWAS.

Supplementary Data 15: Immune-related transcription factors matched with the motif of cis-regulatory elements containing rs10917688.

Supplementary Data 16: Gene set enrichment analysis for transcription factor binding motifs by FUMA-GENE2FUNC.

Supplementary Data 17: Enrichment of active histone marks identified on the rs10917688 by Haploreg.

Supplementary Data 18: Demographic features of the SSc patients of the current study.

Supplementary Data 19: Significantly associated SNPs in each subtype of SSc.

Supplementary Data 20: Association of the FCGR/FCRL variant, rs6697139, in SSc and each subtype.

Supplementary Data 21: Association of TNFAIP3 SNP, rs5029949, with ILD.

Supplementary Table 22: Genetic correlations between SSc and various diseases.

Supplementary Data 23: Genetic correlations between SSc and quantitative traits.

Supplementary Data 24: Tissue-based partitioned heritability analysis for the Japanese and the European dataset (corresponding to Fig. 6A).

Supplementary Data 25: Cell type-based partitioned heritability analysis for Japanese SSc (corresponding to Fig.6B).

Supplementary Data 26: Cell type-based partitioned heritability analysis for European SSc (corresponding to Fig. 6B).

Supplementary Data 27: The output of gchromvar.

Supplementary Data 28: The association of PRSs with SSc susceptibility in Set 1 Japanese dataset based on the best threshold of p-value in each r2 bin.

Supplementary Data 29: Performance of PRS generated using effect sizes of a meta-analysis for the European and Set 1 Japanese dataset (the test result of Set 2).

Supplementary Data 30: Predictive performance of PRS in each subset of SSc.

Supplementary Data 31: Correlation between PRS and age of onset.

Supplementary Data 32: Performance of PRS with or without prioritization of lead SNPs and IRF8-annotated SNPs in RAMOS cells.
