# Supplementary material for: GWAS for systemic sclerosis identifies six novel susceptibility loci including one in the Fcγ receptor region
Source: Nat Commun. 2024 Jan 31;15:319. doi: 10.1038/s41467-023-44541-z (PMC10830486; doi:10.1038/s41467-023-44541-z)
Supplement: Supplementary file 5 — Reporting Summary [file 41467_2023_44541_MOESM5_ESM.pdf]

nature portfolio

Corresponding author(s): Chikashi Terao

Last updated by author(s): Nov 18, 2023

Reporting Summary

Nature Portfolio wishes to improve the reproducibility of the work that we publish. This form provides structure for consistency and transparency in reporting. For further information on Nature Portfolio policies, see our Editorial Policies and the Editorial Policy Checklist.

Statistics

For all statistical analyses, confirm that the following items are present in the figure legend, table legend, main text, or Methods section.

n/a	Confirmed	
☐	☒	The exact sample size (*n*) for each experimental group/condition, given as a discrete number and unit of measurement
☐	☒	A statement on whether measurements were taken from distinct samples or whether the same sample was measured repeatedly
☐	☒	The statistical test(s) used AND whether they are one- or two-sided<br>*Only common tests should be described solely by name; describe more complex techniques in the Methods section.*
☐	☒	A description of all covariates tested
☐	☒	A description of any assumptions or corrections, such as tests of normality and adjustment for multiple comparisons
☐	☒	A full description of the statistical parameters including central tendency (e.g. means) or other basic estimates (e.g. regression coefficient) AND variation (e.g. standard deviation) or associated estimates of uncertainty (e.g. confidence intervals)
☐	☐	For null hypothesis testing, the test statistic (e.g. *F*, *t*, *r*) with confidence intervals, effect sizes, degrees of freedom and *P* value noted<br>*Give P values as exact values whenever suitable.*
☒	☐	For Bayesian analysis, information on the choice of priors and Markov chain Monte Carlo settings
☒	☐	For hierarchical and complex designs, identification of the appropriate level for tests and full reporting of outcomes
☐	☒	Estimates of effect sizes (e.g. Cohen's *d*, Pearson's *r*), indicating how they were calculated

Our web collection on statistics for biologists contains articles on many of the points above.

Software and code

Policy information about availability of computer code

Data collection	All the control samples from BBJ were enrolled from the BioBank Japan (https://biobankjp.org/en/index.html), which is a biobank that collaboratively collects DNA and serum samples from 12 medical institutions in Japan and recruited approximately 200,000 patients with a diagnosis of at least 47 diseases, but not systemic sclerosis. A total of 110,504 samples were enrolled in the present study. For trans-ethnic meta-analysis, the latest GWAS meta-analysis summary data of the European population (PMID: 31672989) was utilized. GTEx ver.7 multi-tissue model consisted of 48 cell/tissue types were utilized for expression quantative loci (eQTL) analysis and Transcriptome-wide association study (TWAS). Partitioned heritability enrichment was also measured in specific cell groups and detailed cell types using the baseline model (ver.2.2). HOMER (http://homer.ucsd.edu/homer/motif/) was utilized for implementing IMPACT.
Data analysis	Quality control for genetic variants and samples were conducted with PLINK1.9. Genotype imputation was conducted with minimac4. The Japanese GWAS was conducted by applying a generalized linear mixed model (GLM) using PLINK2.0 and SAIGE (0.35.8.3). The firth regression was also applied to test the robustness of the significant signals. The trans-ethnic meta-analysis by applying inverse variance-weighed approach using PLINK1.9. GCTA-COJO was utilized for conditional analyses. The statistical finemapping was conducted with the script available at the following website (https://github.com/chr1swallace/finemap-psa/blob/master/bf-functions.R). The regional plots were drawn using LocusZoom software (v1.3).Transcriptome-wide association study (TWAS) was conducted with FUSION software. Functional annotations of given variants, including potential alteration of protein function for exonic variants, were identified by ANNOVAR (version: 2017-07-17). Loss-of-function variants or deleterious exonic variants were explored using VEP/LOFTEE (v1.0.2) or Polyphen2 (v2.2.13) and SIFT (v5.2.2), respectively. The trans-ethnic genetic effect correlation between the European and Japanese was estimated using the python package, Popcorn (ver.0.9.9). Disease-related pathways were explored by FUMA (v1.3.8). Transcription factor binding motif analysis was conducted using Tomtom (v5.3.3). The linkage disequilibrium score regression (LDSC) analysis was conducted using LDSC software (ver.1.0.0). The gchromVAR (v 0.3.0) was utilized with the R code available at the following website (https://github.com/caleblareau/gchromVAR). Polygenic scores (PRS) were calculated using PLINK1.9. The performance of PRS was evaluated by running logistic regression analyses with the area-

under curves and Nagelkerke psuedo-R2 values following the instruction available at the GitHub website (https://choishingwan.github.io/PRS-Tutorial/plink/). IMPACT was utilized by following the instruction available at the website (https://github.com/immunogenomics/IMPACT).

For manuscripts utilizing custom algorithms or software that are central to the research but not yet described in published literature, software must be made available to editors and reviewers. We strongly encourage code deposition in a community repository (e.g. GitHub). See the Nature Portfolio guidelines for submitting code & software for further information.

Data

Policy information about availability of data

All manuscripts must include a data availability statement. This statement should provide the following information, where applicable:

- Accession codes, unique identifiers, or web links for publicly available datasets
- A description of any restrictions on data availability
- For clinical datasets or third party data, please ensure that the statement adheres to our policy

Source data are provided with this paper. The summary statistics is available at figshare (DOI: https://doi.org/10.6084/m9.figshare.23823087). The individual clinical information, such as age, sex, or disease status, is protected and is not available due to data privacy laws.
The following datasets/databases were used for the present study;
GTEx Portal (https://www.gtexportal.org/home), ENCODE (https://www.encodeproject.org), Haploreg (https://pubs.broadinstitute.org/mammals/haploreg/haploreg.php), GWAS catalog (https://www.ebi.ac.uk/gwas/), ImmuNexUT (https://www.immunexut.org), 1000 Genome Project (http://www.1000genomes.org/1000GP: www.1000genomes.org/).
The following databases were used for analyses where applicable;
Regulome DB (https://regulomedb.org/regulome-search/), Tomtom (https://meme-suite.org/meme/tools/tomtom), HOMER (http://homer.ucsd.edu/homer/motif/), VEP/LOFTEE (https://registry.opendata.aws/hail-vep-pipeline/), SIFT (https://sift.bii.a-star.edu.sg/www/publications.html), PolyPhen-2 (http://genetics.bwh.harvard.edu/pph2/index.shtml, gchromVAR (https://github.com/caleblareau/gchromVAR), FUMA (https://fuma.ctglab.nl/)

Research involving human participants, their data, or biological material

Policy information about studies with human participants or human data. See also policy information about sex, gender (identity/presentation), and sexual orientation and race, ethnicity and racism.

Reporting on sex and gender	Both male and female individuals were enrolled in the study. For the sex determination, self-reporting sex and genotype-based sex were compared. We found no discordance between self-reported and genotype-based sex. Due to the higher prevalence of SSc among female, the number of male samples was not enough to conduct the sex-based analysis.
Reporting on race, ethnicity, or other socially relevant groupings	The individuals included in the SSc-GWAS were all Japanese. For the trans-ethnic meta-analysis, we used summary statistics of the latest European meta-GWAS summary statistics derived from 14 independent epidemiological cohorts of European ancestries.
Population characteristics	The characteristics of Japanese subjects are provided in Supplementary Data 18. The population characteristics of the European samples are found in the original manuscript (doi: 10.1038/s41467-019-12760-y).
Recruitment	The Japanese case samples were enrolled from a total of 23 centers in Japan. From Biobank Japan (BBJ), we selected 110,504 subjects as control. Additional 2,105 were enrolled from a single center in Japan.
Ethics oversight	The study was approved by the Ethics Committee of RIKEN (17-17-16(16))

Note that full information on the approval of the study protocol must also be provided in the manuscript.

Field-specific reporting

Please select the one below that is the best fit for your research. If you are not sure, read the appropriate sections before making your selection.

[x] Life sciences [ ] Behavioural & social sciences [ ] Ecological, evolutionary & environmental sciences

For a reference copy of the document with all sections, see nature.com/documents/nr-reporting-summary-flat.pdf

Life sciences study design

All studies must disclose on these points even when the disclosure is negative.

Sample size	A total of 1,499 cases and a total of 112,609 controls were enrolled. This was the largest dataset for Japanese SSc GWAS ever.
Data exclusions	71 cases and 10 controls were excluded after sample quality controls to avoid any spurious results.
Replication	The Japanese GWAS (a combined dataset of Set1 and Set2) was compared with that previously conducted with smaller sample size (Set1, PMID: 28314753) and a dataset consisting of newly enrolled subjects (Set 2), and confirmed comparable results among the datasets. We also confirmed the robustness of the GWAS signals in firth regression and a random-effect model. We conducted the trans-ethnic meta-analysis and observed that most of the signals previously identified the European GWAS meta-analysis were replicated. The analyses were repeated at least two times to confirm the robustness of the results.

Randomization	This is a case-control study and sample randomization is not applicable. All the cases and controls were enrolled from multiple centers throughout Japan. The Set1 and Set2 datasets are determined by the time of the enrollment and the genotyping chips utilized, which could be confounding factors.
Blinding	All individual data were anonymized to protect privacy of the participants.

Reporting for specific materials, systems and methods

We require information from authors about some types of materials, experimental systems and methods used in many studies. Here, indicate whether each material, system or method listed is relevant to your study. If you are not sure if a list item applies to your research, read the appropriate section before selecting a response.

Materials & experimental systems

n/a	Involved in the study
☒	☐ Antibodies
☒	☐ Eukaryotic cell lines
☒	☐ Palaeontology and archaeology
☒	☐ Animals and other organisms
☒	☐ Clinical data
☒	☐ Dual use research of concern
☒	☐ Plants

Methods

n/a	Involved in the study
☒	☐ ChIP-seq
☒	☐ Flow cytometry
☒	☐ MRI-based neuroimaging

Plants

Seed stocks	*Report on the source of all seed stocks or other plant material used. If applicable, state the seed stock centre and catalogue number. If plant specimens were collected from the field, describe the collection location, date and sampling procedures.*
Novel plant genotypes	*Describe the methods by which all novel plant genotypes were produced. This includes those generated by transgenic approaches, gene editing, chemical/radiation-based mutagenesis and hybridization. For transgenic lines, describe the transformation method, the number of independent lines analyzed and the generation upon which experiments were performed. For gene-edited lines, describe the editor used, the endogenous sequence targeted for editing, the targeting guide RNA sequence (if applicable) and how the editor was applied.*
Authentication	*Describe any authentication procedures for each seed stock used or novel genotype generated. Describe any experiments used to assess the effect of a mutation and, where applicable, how potential secondary effects (e.g. second site T-DNA insertions, mosiacism, off-target gene editing) were examined.*
