# Supplementary material for: Author Correction: GWAS for systemic sclerosis identifies six novel susceptibility loci including one in the Fcγ receptor region
Source: Nat Commun. 2026 Jan 19;17:617. doi: 10.1038/s41467-026-68405-4 (PMC12816564; doi:10.1038/s41467-026-68405-4)
Supplement: Supplementary file 1 — Original and revised Table 1 [file 41467_2026_68405_MOESM1_ESM.pdf]

Table 1. Genome-wide significant SNPs identified in GWAS of Japanese SSc.

The original table

							Combined dataset (Set 1 and Set 2)					Set 1			Set 2			EUR			
CHR	POS	ID	TYPE	GENE	EA	NEA	EAF (case control)		BETA	SE	P-value	BETA	SE	P-value	BETA	SE	P-value	EAF	BETA	SE	P-value
**1**	**161660696**	**rs6697139**	**intergenic**	***RPL31P11-FCRLA***	**T**	**G**	**0.038**	**0.024**	**0.312**	**0.199**	**4.93E-11**	**0.316**	**0.208**	**4.85.E-04**	**0.32**	**0.15**	**7.90E-07**	**0.225**	**0.026**	**0.037**	**0.1107**
2	191943742	rs11889341	intronic	*STAT4*	T	C	0.373	0.299	0.161	0.040	2.51E-20	0.187	0.069	3.62E-10	0.14	0.056	7.57E-09	0.229	0.127	0.094	0.0018
**6**	**138197506**	**rs5029949**	**intronic**	***TNFAIP3***	**G**	**A**	**0.102**	**0.07**	**0.175**	**0.063**	**1.66E-10**	**0.183**	**0.114**	**2.03E-04**	**0.18**	**0.087**	**1.12E-06**	**0.022**	**0.126**	**0.085**	**0.0007**
7	128575797	rs1450734198	intergenic	*KCP-IRF5*	TCTTAGCTATTGCTC	T	0.163	0.127	0.15	0.055	2.74E-10	0.089	0.097	0.0346	0.19	0.074	2.40E-09	NA	NA	NA	NA
**14**	**105408955**	**rs2819422**	**exonic‡**	***AHNAK2***	**G**	**A**	**0.312**	**0.369**	**-0.12**	**0.041**	**2.73E-11**	**-0.143**	**0.069**	**1.55E-06**	**-0.12**	**0.058**	**2.84E-06**	**0.466**	**0.04**	**0.035**	**0.0092**

Corrected table (red highlighted)

							Combined dataset (Set 1 and Set 2)					Set 1			Set 2			EUR			
CHR	POS	ID	TYPE	GENE	EA	NEA	EAF (case control)		BETA	SE	P-value	BETA	SE	P-value	BETA	SE	P-value	EAF	BETA	SE	P-value
**1**	**161660696**	**rs6697139**	**intergenic**	***RPL31P11-FCRLA***	**T**	**G**	**0.038**	**0.024**	**0.719**	**0.109**	**4.93E-11**	**0.727**	**0.208**	**4.85.E-04**	**0.74**	0.15	**7.90E-07**	**0.225**	**0.059**	0.037	**0.1107**
2	191943742	rs11889341	intronic	*STAT4*	T	C	0.373	0.299	0.37	0.040	2.51E-20	0.432	0.069	3.62E-10	0.33	0.056	7.57E-09	0.229	0.292	0.094	0.0018
**6**	**138197506**	**rs5029949**	**intronic**	***TNFAIP3***	**G**	**A**	**0.102**	**0.07**	**0.402**	**0.063**	**1.66E-10**	**0.422**	**0.114**	**2.03E-04**	**0.423**	0.087	**1.12E-06**	**0.022**	**0.291**	**0.085**	**0.0007**
7	128575797	rs1450734198	intergenic	*KCP-IRF5*	TCTTAGCTATTGCTC	T	0.163	0.127	0.346	0.055	2.74E-10	0.206	0.097	0.0346	0.44	0.074	2.40E-09	NA	NA	NA	NA
**14**	**105408955**	**rs2819422**	**exonic‡**	***AHNAK2***	**G**	**A**	**0.312**	**0.369**	**-0.276**	**0.041**	**2.73E-11**	**-0.33**	**0.069**	**1.55E-06**	**-0.27**	0.058	**2.84E-06**	**0.466**	**0.092**	**0.035**	**0.0092**

1,428 cases and 112,599 controls were analyzed by logistic regression. EUR, European; CHR, chromosome; POS, genomic position in GRCh37 coordinate; EA, effect allele; NEA non-effect allele; EAF, effect allele frequency; BETA, beta coefficient of logistic regression; SE, standard error;

‡ nonsynonymous SNV AHNAK2:NM_001350929:exon7:c.T12533C:p.V4178A,AHNAK2:NM_138420:exon7:c.T12833C:p.V4278A; gene symbols are indicated in italics; the novel disease-associated SNPs are highlighted with bold
